# SNHG3 Affects Gastric Cancer Development by Regulating SEPT9 Methylation

**DOI:** 10.1155/2022/3433406

**Published:** 2022-04-28

**Authors:** Wei Li, Xudong Ma, Feng Wang, Shi Chen, Qingxiong Guo, Feng Sun, Yongqing Duan

**Affiliations:** ^1^Department of Blood Transfusion, The Second Affiliated Hospital of Kunming Medical University, Kunming, China; ^2^Department of Gastrointestinal Surgery, The Second Affiliated Hospital of Kunming Medical University, Kunming, China

## Abstract

**Background:**

Gastric cancer (GC) is a common malignancy that can be formed by methylation-induced deactivation of tumor silencer genes, which is one of the key mechanisms of tumorigenesis. SEPT9 methylation, a symptomatic marker for tumors, can downregulate gene expression. Long noncoding RNA small nucleolar host gene 3 (lncRNA SNHG3) is a new type of lncRNA related to cancer. Our study investigated the mechanism of SNHG3 regulation of SEPT9 methylation and its effects on the growth, metastasis, and spread of gastric cancer cells.

**Methods:**

Quantitative real-time PCR (qRT–PCR) was used to detect SNHG3 and miR-448 in gastric cancer, and a dual-luciferase experiment verified the effects of SNHG3, miR-448, and DNMT1. After abnormally expressing SNHG3, miR-448, and DNMT1 alone or together, methylation-specific PCR was performed to determine the methylation of SEPT9, Western blotting was performed to detect the expression of DNA methyltransferase 1 (DNMT1) and SEPT9, and Transwell, scratch, and CCK-8 assays were performed to reveal the invasion, migration, and cell growth of gastric cancer cells.

**Results:**

We found that SNHG3 was upregulated in gastric cancer and that SNHG3 knockdown or miR-448 overexpression inhibited SEP9 methylation and therefore increased its expression, thereby inhibiting the growth, metastasis, and spread of gastric cancer cells.

**Conclusion:**

Our study indicates that SNHG3 regulates SEPT9 methylation by targeting miR-448/DNMT1 and subsequently affecting the occurrence and development of gastric cancer.

## 1. Introduction

Gastric cancer (GC) is an common malignant tumor and the second most deadly tumor in the world. Although great improvements have been achieved in recent years in treatment strategies, such as surgery, radiotherapy, and adjuvant chemotherapy [1–3], the prognosis of patients with advanced gastric cancer remains poor, which is closely related to the diagnostic stage of the disease; therefore, new early diagnostic models and new curative approaches are urgently needed. DNA methylation is a common method of modifying genomic DNA, and tumor suppressor gene deactivation caused by methylation is one of the key mechanisms in the development of tumors [4–6]. A number of studies have confirmed that aberrant DNA methylation disrupts the normal expression and function of multiple genes involved in tumor regulation, thereby affecting tumorigenesis and progression [7, 8]. For example, Pinin induces epithelial to mesenchymal transition in hepatocellular carcinoma by regulating m6A modification, which leads to the malignant progression of hepatocellular carcinoma. The effect of DNA methylation modification on the progression of gastric cancer has also been reported; for example, DNA methylation mediates the downregulation of miR-33b expression in gastric cancer, thereby weakening the role of miR-33b as a tumor suppressor. ALKBH5 promotes the invasion and transformation of gastric cancer by reducing the methylation of the lncRNA NEAT1.

SEPT9 is an evolutionarily highly conserved skeletal protein widely found in eukaryotes. It is an important component of the cytoskeleton [9] that influences cell polarization, intracellular material transport, extracellular secretion, cell cycle regulation, and cell apoptosis. SEPT9 methylation leads to the downregulation of gene expression and affects the process of rectal cancer, prostate cancer, and breast cancer; therefore, it can be used as an indicator of tumors [10–12]. However, the connection between SEPT9 methylation and gastric cancer expansion still needs to be further explored. Consequently, the connection between SEPT9 methylation and gastric cancer development and the related infinitesimal mechanisms that regulate SEPT9 methylation must be investigated. DNA methyltransferase 1 (DNMT1) is a multiregional protein that influences the establishment and regulation of the tissue-specific pattern of methylated cytosine residues [13], and it can mediate epigenetic suppression and result in tumorigenesis and progression [14, 15]. In addition, tumor progression can be inhibited by reversing DNA methylation and restoring the tumor suppressor genes silenced by methylation through the inhibition of methyltransferase activity.

LncRNAs are a heterogeneous variation of noncoding RNAs that are widely distributed in the genome and have a length of more than 200 nt, and they have an influence on regulating cell growth, apoptosis, invasion, and epithelial mesenchymal transition (EMT) [16–19]. The competing endogenous RNA (ceRNA) hypothesis suggests that lncRNAs have miRNA sites and bind to miRNAs, thereby indirectly governing the expression of miRNA target genes. Small nucleolar RNA host gene 3 (SNHG3) is a new type of lncRNA related to liver cancer, colorectal cancer, laryngeal cancer, and other cancers, and it is considerably upregulated in hepatocellular carcinoma and promotes cell invasion and epithelial-mesenchymal transition through the miR-128/CD151 pathway [20]. Huang et al. found that SNHG3 regulates miR-182-5p release and c-Myc expression to promote colorectal cancer [21]. However, the role of SNHG3 and its regulated miRNAs in the course of gastric cancer has not been elucidated.

In a previous study, miR-448 expression was found to be reduced in gastric cancer [22]; however, miR-448 overexpression has been shown to inhibit gastric cancer cell growth, clone constitution, and invasion. In this study, bioinformatics database TargetScan was used to predict the 3′UTR of SNHG3 as a target of miR-448. Therefore, we speculate that SNHG3 may regulate SEPT9 methylation through miR-448, thereby regulating the progression of gastric cancer. We knocked out SNHG3 to inhibit the expression level of DNMT1 by regulating miR-448, which inhibits the methylation of SEPT9 mediated by DNMT1, upregulates the expression level of SEPT9, and prevents the invasion, metastasis, and spread of gastric cancer cells.

## 2. Materials and Methods

### 2.1. Clinical Sample

Tissues from 25 gastric tumor and corresponding nontumor cases were collected from 2016 to 2019 in the Department of Gastroenterology of our hospital. Samples were stored in liquid nitrogen for further analysis after surgical removal of the desired tissue. Informed consent was obtained for each patient. This study was approved by the Medical Ethics Committee of the Second Affiliated Hospital of Kunming Medical University (Shen-PJ-2021-17).

### 2.2. Cell Culture

The human gastric tumor cell lines HGC-27 and MGC-803 and the human gastric mucosal cell line GES-1 were purchased from Procell (Wuhan, China). Cells were cultivated in RPMI-1640 medium containing 10% fetal bovine serum, 100 U/mL penicillin, and 100 *μ*g/mL streptomycin (Thermo Fisher, Waltham, MA, USA) and maintained in an environment with 5% carbon dioxide.

### 2.3. Quantitative Real-Time PCR (qRT–PCR)

Total RNA was extracted from tissues and cells using TRIzol (Invitrogen, Carlsbad, CA, USA) reagent. Two micrograms of RNA was reverse-transcribed into cDNA using a Reverse transcription kit (Invitrogen, Carlsbad, CA, USA). Two microliters of the reverse-transcription products was extracted for a PCR assay, and GAPDH and U6 were used as internal reference genes. The primer sequences were as follows: SNHG3 F: 5′-TTCAAGCGATTCTCGTGCC-3′, R: 5′-AAGATTGTCAAACCCTCCCTGT-3′; miR-488 F: 5′-CGGGGCAGCUCAGUACAG-3′, R: 5′-CAGTGCGTGTCGTGGAGT-3′; GAPDH F: 5′-AATGGGCAGCCGTTAGGAAA-3′, R: 5′-TGAAGGGGTCATTGATGGCA-3′; U6 F: 5′-CTCGCTTCGGCAGCACA-3′, R: 5′-AACGCTTCACGAATTTGCGT-3′. Real-time polymerase chain reaction was performed according to the instructions of the SYBR Master Mix kit (Thermo Fisher, Waltham, MA, USA). The detection result was calculated by the 2^−ΔΔCt^ method.

### 2.4. Western Blotting (WB)

Total cell protein was extracted with cell lysate RIPA buffer containing protease inhibitors, the protein concentration was detected by BCA Protein Assay Kit (Beyotime, P0012), and the protein was denatured at 100°C for 5 min. SDS–PAGE was used to transfer the protein to a PVDF membrane after electrophoresis. Then, the corresponding primary antibodies against DNMT1, SEPT9, Snail2, and MMP9 (1 : 1000, Abcam, Cambridge, MA, USA) were added and incubated overnight at 4°C (using *β*-actin as an internal control). On the second day, HRP-labelled antibody (1 : 1000, Abcam, Cambridge, MA, USA) was added and incubated at room temperature for 1.5 h. Finally, after adding ECL solution, the gray values of the bands were calculated by ImageJ (National Institutes of Health; 1.8.0 version).

### 2.5. Dual-Luciferase Reporter Gene Assay

The 3′UTRs of SNHG3 and DNMT1 were synthesized and cloned into the pmirGLO dual-luciferase reporter vector to construct pmirGLO-SNHG3-WT/MUT and pmirGLO-DNMT1-WT/MUT recombinant plasmids. Then, cotransfection with the miR-448 mimic or NC-mimic into HEK 293T cells and cellular luciferase activities were detected according to the instructions of the dual-luciferase reporter gene detection kit (Promega, Madison, WI, USA).

### 2.6. Methylation-Specific PCR (MSP)

Cell DNA was extracted with a kit (Invitrogen, Carlsbad, CA, USA) and then treated with sodium bisulfite, and a PCR assay was performed. The PCR products were stained with ethidium bromide, subjected to 2% agarose gel electrophoresis, and observed under ultraviolet light.

### 2.7. Cell Counting Kit-8 (CCK-8)

Cell growth was measured using a CCK-8 kit (AbMole, Shanghai, China). The cells were collected and seeded at 2000 cells/well in a 96-well plate, and the reaction solution was added and incubated at 37°C for 4 h. Cell proliferation was detected at 48 hours. The optical density was measured at an absorbance value of 450 nm.

### 2.8. Transwell Assay

For the Transwell assays, which measure cell invasion, the cells were trypsinized and located in the higher chamber. Then, the cells were fixed and stained with 0.1% crystal violet after incubation at 37°C for 24 h, and then, the number of invaded cells was counted.

### 2.9. Scratch Assay

For the scratch assays, which measure cell migration, cells grown during the logarithmic growth phase were selected after transfection and seeded in 6-well plates, and a pipette tip was used to scratch parallel lines on a vertical 6-well plate, which was washed twice with PBS to wash away multiple cells. After culturing for 24 hours, cell mobility was observed and measured.

### 2.10. Statistical Analysis

GraphPad Prism 7.0 (Inc, San Diego, CA, USA) was used to analyze the experimental data, which are expressed as the mean ± standard deviation (SD), and draw related figures. *T* tests were performed for comparisons between the two groups, and one-way ANOVA was used for comparisons between several groups. Differences were statistically significant at *p* < 0.05. Each experiment was repeated three times.

## 3. Results

### 3.1. SNHG3 Upregulation and miR-448 Downregulation in Gastric Cancer Samples

**Figure 1:**
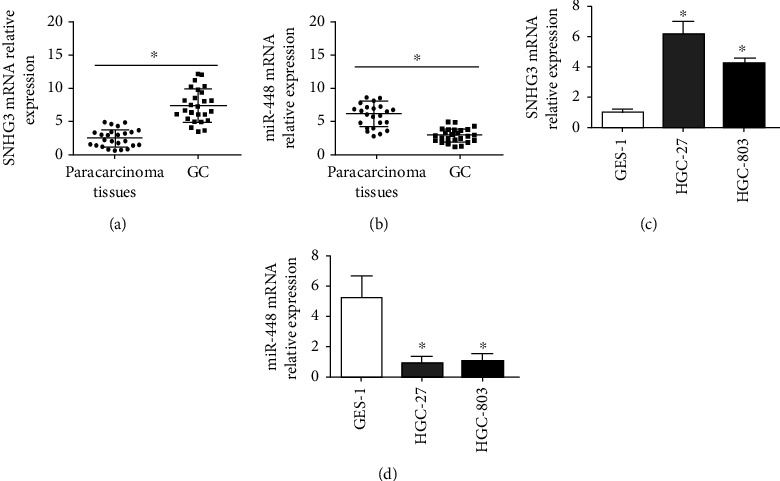
SNHG3 and miR-448 are abnormally expressed in GC tissues and cells. (a) qRT–PCR showing the differences in SNHG3 in GC tissues and paracarcinoma tissues and (b) qRT–PCR showing the differences in miR-448 in GC tissues and paracarcinoma tissues. ^∗^*p* < 0.05 based on comparison with the NC group. (c) qRT–PCR showing the differences in SNHG3 expression in gastric cancer cells and normal intestinal epithelial cells and (d) qRT–PCR showing the differences in miR-448 expression in gastric cancer cells and normal intestinal epithelial cells. ^∗^*p* < 0.05 based on comparisons with the GES-1 group.

To analyze the function of SNHG3 and miR-448 in gastric cancer, qRT–PCR was performed to assess the expression of SNHG3 and miR-448 in 25 cases. The results demonstrated that SNHG3 was upregulated, and miR-448 was downregulated in the gastric cancer tissue samples (Figures 1(a) and 1(b)). In addition, we also performed similar assays to detect the expression of SNHG3 and miR-448 in the gastric cancer cell lines HGC-27 and MGC-803 and the gastric mucosal cell line GES-1. The results showed that SNHG3 was higher in the gastric cancer cell lines HGC-27 and MGC-803 than in the gastric mucosal cell line GES-1 and miR-448 and was higher in GES-1 than in HGC-27 and MGC-803 (Figures 1(c) and 1(d)).

### 3.2. Knockout of SNHG3 Affects the Methylation of SEPT9 in Gastric Cancer Cells

**Figure 2:**
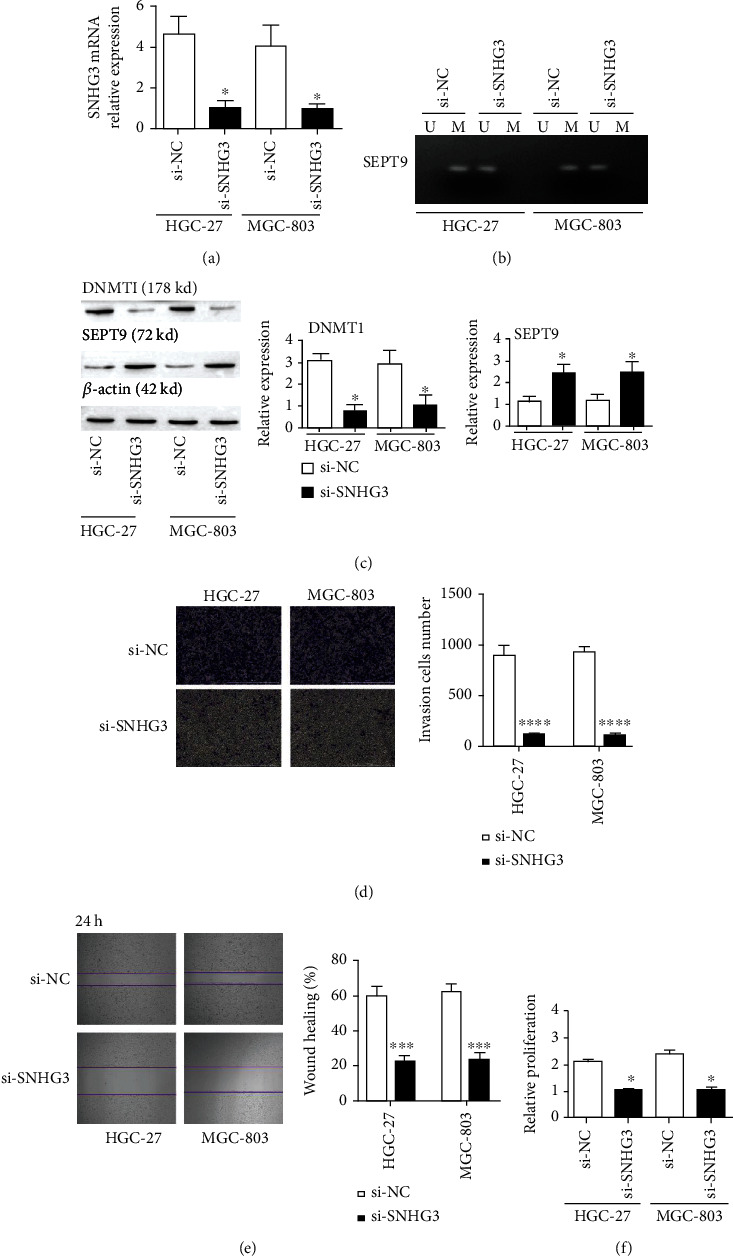
SNHG3 knockout inhibits SEPT9 methylation and malignant cytological behavior of GC cells. (a) qRT–PCR detection of the expression level of SNHG3; (b) MSP detection of the methylation level of SEPT9; (c) WB detection of the expression of DNMT1 and SEPT9; (d) Transwell assay detection of the invasion ability of GC cells, scale bar = 1000 *μ*m; (e) scratch assay detection of the migration ability of GC cells; and (f) CCK-8 detection of cell growth activity. ^∗^*p* < 0.05 based on comparisons with the si-NC group.

To explore how SEPT9 methylation is regulated by SNHG3 in gastric cancer, we transfected SNHG3-specific siRNA (si-SNHG3) or control siRNA (si-NC) into the GC cell lines HGC-27 and MGC-803. qRT–PCR revealed that SNHG3 mRNA expression decreased after si-SNHG3 transfection (Figure 2(a)). Methylation-specific PCR detected methylation of SEPT9 and high levels of methylation in the GC cell lines HGC-27 and MGC-803; moreover, methylation disappeared after knocking out SNHG3 (Figure 2(b)). In addition, SI-SNHG3 transfection inhibited DNMT1 but activated SEPT9 (Figure 2(c)). We performed Transwell and scratch assays to analyze the cell dispersal and metastasis, and they revealed that the reduction in SNHG3 expression considerably inhibited the dispersal and metastasis of GC cells (Figures 2(d) and 2(e)). The CCK-8 assay proved that SNHG3 knockout suppressed the growth of gastric cancer cells (Figure 2(f)). The above results explain why SNHG3 knockout inhibits SEPT9 methylation of gastric cancer cells as well as the migration, invasion, and proliferation of these cells.

### 3.3. SNHG3 Targets miR-448/DNMT1 to Regulate Gastric Cancer Cells

**Figure 3:**
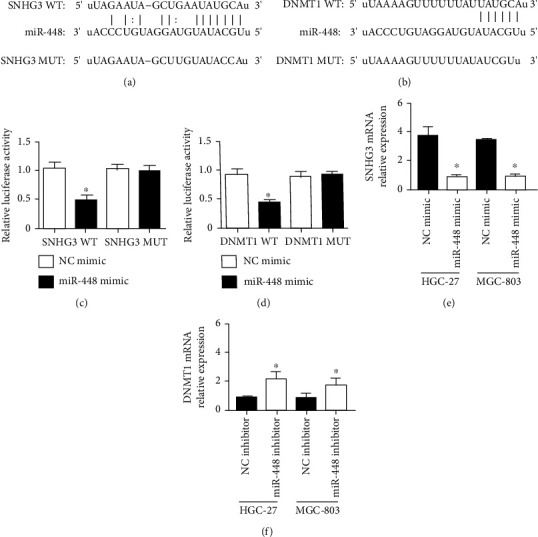
Targeting correlation between SNHG3 and miR-448 and between DNMT1 and miR-448. (a) Binding site between SNHG3 and miR-448; (b) binding site between miR-448 and DNMT1; (c) dual-luciferase reporter gene experiment to evaluate the luciferase activity of transfected NC mimic/miR-448 mimic and SNHG3 WT/MUT; (d) dual-luciferase gene experiment to evaluate the luciferase activity of transfected NC mimic/miR-448 mimic and DNMT1 WT/MUT; (e) qRT–PCR detection of the expression of SNHG3 after transfection of the NC mimic/miR-448 mimic; (f) qRT–PCR detection of the expression of DNMT1 after transfection of the NC inhibitor/miR-448 inhibitor. ^∗^*p* < 0.05 based on comparisons with the NC group.

To study the connections among SNHG3, miR-448, and DNMT1, we applied the bioinformatics software TargetScan to predict the correlations between SNHG3 and miR-448 and between DNMT1 and miR-448 (Figures 3(a) and 3(b)). In addition, we found that miR-448 mimics cotransfected with plasmids inhibited the relative luciferase activity of SNHG3/DNMT1 WT but not that of SNHG3/DNMT1 MUT (Figures 3(c) and 3(d)). SNHG3 mRNA expression in HGC-27 and MGC-803 cells was suppressed by miR-448 overexpression, and inhibition of miR-448 increased the expression level of DNMT1 mRNA. The results suggest that SNHG3 can serve as a molecular scaffold for miR-448 targeting DNMT1 (Figures 3(e) and 3(f)).

### 3.4. SNHG3 Promotes SEPT9 Methylation in Gastric Cancer Cells via miR-448

**Figure 4:**
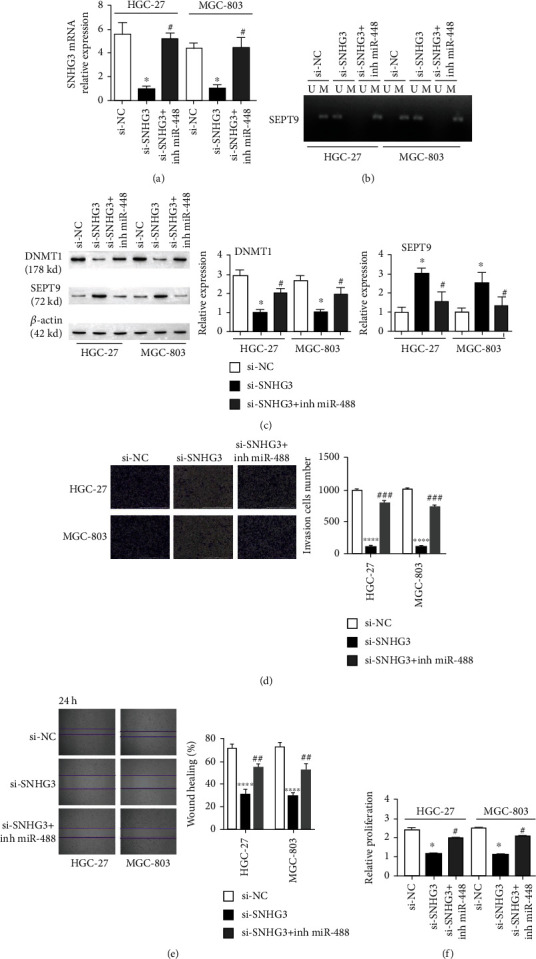
Inhibiting miR-448 eliminated the effect of si-SNHG3 transfection on gastric cancer cells. (a) qRT–PCR detection of the expression level of SNHG3; (b) MSP detection of the methylation level of SEPT9; (c) WB detection of the expression of DNMT1 and SEPT9; (d) Transwell assay detection of the invasion ability of GC cells, scale bar = 1000 *μ*m; (e) scratch assay detection of t migration ability of GC cells; and (f) CCK-8 detection of cell proliferation activity. ^∗^*p* < 0.05 compared with the si-NC group.

To corroborate the function of SNHG3 targeting miR-448 in gastric cancer cells, we inhibited miR-448 (inh miR-448) in cells transfected with si-SNHG3. The qRT–PCR results showed that the mRNA expression of SNHG3 decreased after inhibiting SNHG3; moreover, this situation changed after inhibiting SNHG3 and miR-448 (Figure 4(a)). Furthermore, methylation-specific PCR and WB results illustrated that SEPT9 methylation increased and SEPT9 expression decreased after miR-448 inhibition (Figures 4(b) and 4(c)). The expression of DNMT1 decreased after transfection with si-SNHG3 and increased after inhibition of miR-448 (Figure 4(c)). The Transwell, scratch, and CCK-8 assays showed that simultaneous inhibition of miR-448 and SNHG3 can promote cell invasion, migration, and proliferation (Figures 4(d)–4(f)). These observations revealed that SNHG3 targets the downregulation of miR-448 and that miR-448 inhibition reverses changes in the methylation status and the growth, migration, and invasion of gastric cancer cells after SNHG3 knockout.

### 3.5. miR-448/DNMT1 Regulates SEPT9 Methylation and Malignant Cytology of Gastric Cancer Cells

**Figure 5:**
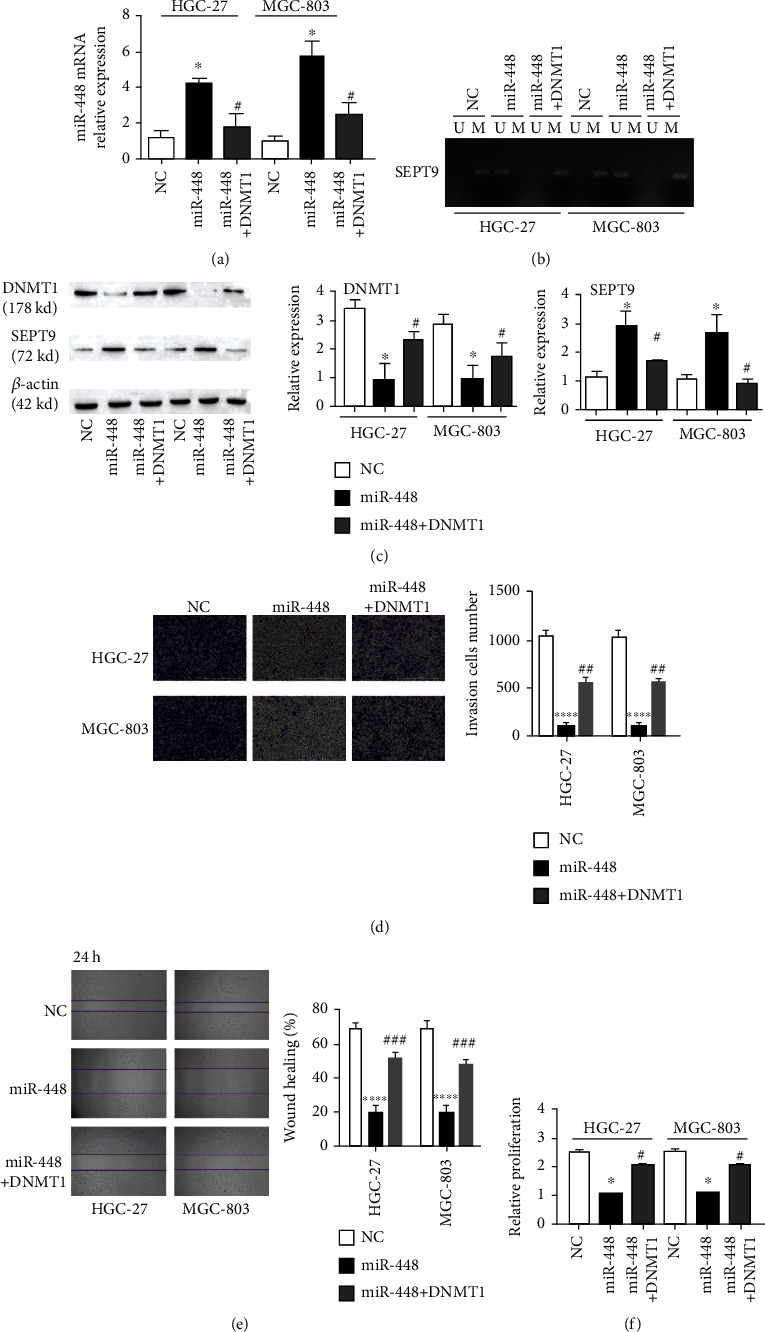
miR-448 regulates SEPT9 methylation, invasion, migration, and proliferation through DNMT1. (a) qRT–PCR detection of the expression of SNHG3; (b) MSP detection of the methylation level of SEPT9; (c) WB detection of the expression of DNMT1 and SEPT9; (d) Transwell assay detection of the invasion ability of GC cells, scale bar = 1000 *μ*m; (e) scratch assay detection of the migration ability of GC cells; and (f) CCK-8 detection of cell growth activity. ^∗^*p* < 0.05 compared with the NC group.

HGC-27 and MGC-803 cell lines were transfected with the miR-448 mimic and NC and subjected to DNMT1 overexpression to determine the effect of miR-448/DNMT1 on adjusting SEPT9 methylation in bladder cancer cells. Transfection of the miR-448 mimic upregulated the expression of miR-448 increased, while overexpression of miR-448 and DNMT1 downregulated the expression of miR-448 (Figure 5(a)). The MSP assay showed that the increase in miR-448 resulted in the suppression of SETP9 methylation (Figure 5(b)). Western blotting verified the expression levels of DNMT1 and SEPT9. Overexpression of miR-448 reduced the expression of DNMT1 and increased the expression of SEPT9, while overexpression of miR-448 and DNMT1 reversed this effect (Figure 5(c)). Transwell, scratch, and CCK-8 assays demonstrated that DNMT1 overexpression promoted the dispersal, metastasis, and growth of gastric cancer cells (Figures 5(d)–5(f)). This finding indicates that overexpression of DNMT1 restores the miR-488-inhibited methylation of SEPT9, thereby promoting cell migration, invasion, and proliferation.

## 4. Discussion

GC is a malicious tumor that originates from the epithelial cells of the gastric mucosa on the inner surface of the stomach wall and affects various parts of the stomach, and its mortality and morbidity have been consistently high [23, 24]. Consequently, knowledge of the molecular mechanism of gastric cancer is very important for identifying new diagnosis and treatment options. DNA methylation is among several methods of DNA modification and represents a warning marker for cancer based on the selective addition of methyl groups to DNA under the catalysis of DNA methyltransferase (DNMT). Studies have found that SEPT9 methylation can be used as a diagnostic marker for tissue or organ disease [25]. Few studies have reported the role of SEPT9 methylation in gastric cancer; thus, this study investigated the regulatory mechanism of SEP9 methylation in gastric cancer.

A number of studies have shown that lncRNAs and miRNAs are involved in tumorigenesis and development. For example, lncRNA XLOC_006390 can be used as a ceRNA to inversely regulate the expression of miR-331-3p and miR-338-3p, thereby encouraging cervical carcinogenesis and metastasis [26, 27]. These results showed that the reciprocity between lncRNAs and miRNAs is very important in cancer. SNHG3 is a member of a lncRNA family that plays a leading role in a variety of cancers, including liver, colorectal, and bladder cancers [28–30]. However, the mechanism by which SNHG3 influences GC has not been revealed. Our study found that lncRNA SNHG3 was highly expressed in GC. Silencing SNHG3 inhibited the expression of DNMT1 and SEPT9 methylation, and SEPT9 expression was upregulated and inhibited gastric cancer cell growth, metastasis, and spread.

We further explored the mechanism underlying the ability of SNHG3 to affect SEPT9 methylation. Bioinformatics software was used to forecast the complementary sites between SNHG3 and miR-448, and we revealed that DNA methyltransferase 1 (DNMT1) and miR-448 also have binding sites. Dual-luciferase reporter experiments combined with qRT–PCR proved that SNHG3 targets and regulates miR-448/DNMT1. Furthermore, we inhibited the expression of miR-448 in the GC cell lines HGC-27 and MGC-803 transfected with si-SNHG3. The results proved that miR-448 can interact with SNHG3 to participate in SEPT9 methylation, inhibit miR-448 to promote SEPT9 methylation, and restore gastric cancer cell growth, metastasis, and spread. Then, we transfected miR-448 mimics into GC cells. Upregulation of miR-448 promotes SEP9 protein expression and inhibits the growth, metastasis, and spread of bladder cancer cells, and these processes are reversed by the overexpression of DNMT1. This finding further shows that miR-448 regulates SEPT9 methylation and its expression through DNMT1.

**Figure 6:**
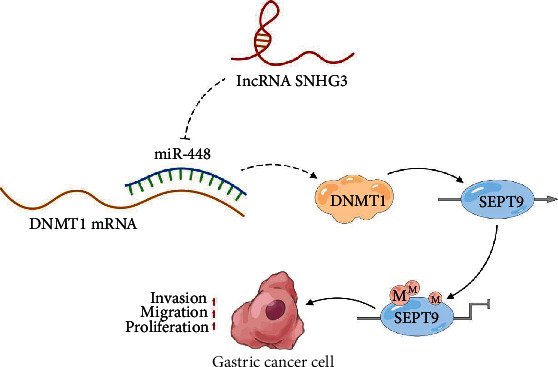
A schematic model of SNHG3/miR-448/DNMT1 molecular axis mediated SEP9 methylation to promote gastric cancer progression.

In conclusion, our study showed that lncRNA SNHG3 is highly expressed in gastric cancer tissues and cells. Knockdown of lncRNA SNHG3 upregulates miR-448 and suppresses DNMT1 expression, thereby inhibiting DNMT1-mediated SEP9 methylation and upregulating SEP9 expression to suppress gastric cancer progression (Figure 6). Thus, the SNHG3/miR-448/DNMT1 molecular axis could provide new insights for understanding the mechanisms by which methylation affects the progression of GC and facilitate the identification of new diagnostic and therapeutic targets. Although we confirmed in vitro that the SNHG3/miR-448/DNMT1 molecular axis mediates SEP9 methylation and thus affects the progression of gastric cancer, this study was limited in its ability to further verify this molecular mechanism in vivo. In addition, SEP9 expression and methylation were not detected in clinical samples. In the future, we plan to build an animal model to verify this molecular mechanism and increase the detection of various indicators in the molecular axis of gastrointestinal cancer tissues and in serum samples to clarify their specific roles as markers of gastric cancer.

## Data Availability

The data used to support the findings of this study are included within the article.
